# It Smells to Heaven

**Published:** 1888-12

**Authors:** 


THE following editorial which appeared in our October number, attracted very wide attention, and we received orders for *over one hundred copies more than we could fill.*

At the request of several of our correspondents, and to accommodate certain parties in New York and Cincinnati whose orders we were unable to fill, the article is here reproduced; and opportunely, we hope, inasmuch as an interesting letter in reply thereto, by Dr. Cunningham, appears simultaneously in this issue. We beg the indulgence of our subscribers in republishing the article, but the facts stated above, the great importance of the subject, the universal interest its publication awakened, together with the good which we hope will be accomplished by thus directing attention to the importance of *food inspection*, constitute our justification in the departure from our usual custom. Legislation is badly needed in the direction of preventing food adulteration and protecting the public from such practices as are mentioned in the article.—Ed.

IT SMELLS TO HEAVEN.

"There are reasons to believe that Moses was an 'advanced sanitarian,' and had a knowledge of *trichina spiralis*, and *cysticerci*, in the swine; (indeed, these parasites are found to-day, in the wild boar of Palestine;) and it has been said that he forbade the eating of pork on sanitary grounds, and only gave the command a religious impress the better to enforce it. *Per contra*, an old Jewish gentleman of our acquaintance says Moses was an advanced thinker on other subjects, and had an eye to business; that Aaron, having purchased all the pork in the market, ("got a corner on it," as it were,) refused to let Moses participate in the 'spec.' Moses then quietly purchased all the beef cattle to be had, and *then* issued his order; thus 'busting Aaron up,' says our friend.

"Whatever may have been the reasons which induced Moses to forbid the eating of pork, Moses is entitled to the undying gratitude of the Jewish race; and it is a great pity that the command could not have been extended to gentile as well, and enforced to this day.

"We have always regarded the eating of pork, or the 'product of the hog' in any shape, as a great sanitary evil; and though not a Jew, we have strong convictions that the use of hog's flesh, or any part of the animal for human food, is deleterious to the general health, and should be prohibited. The decision of the Congress of Tuberculosis in Paris, recently rendered, to the effect that consumption is most frequently acquired from the meat or milk of animals, (pork, beef and milk), renders the discussion ot this subject peculiarly opportune at this time.

"Aside from any consideration of infection with *trichina* or *cysticerci* (the great consideration), the hog is 'unclean,' in every sense; he is a scavenger; he wallows in filth, and disputes the possession of the offal of the slaughter pens with the buzzard, and fights over the carcass of the dead horse, robbing the maggots of their legitimate prey! He revels in filth, and devours even human excrement! Who would eat a buzzard? or a cat? Yet these are *clean*, compared to the swine. They are filthy within and without; and, according to a late sanitary officer in Cincinnati, writing in the *Lancet and Clinic*, the entrails of every hog are occupied by the large round, (lumbricoid), or some other kind of worm! And yet, civilized man *eats hog's flesh knowingly*; and—*unknowingly*, eats—the Lord only knows what, as we shall presently explain.

"Congress has recently investigated the manufacture of the lard of commerce, so-called 'choice,' 'prime' and 'leaf;' and some of the same men who worked at the tanks testified under oath that the lard was made from 'the guts, and the head and the feet' of the hog, put in the tanks along with other parts; anything and everything that contained a cell of adipose; moreover, that these,—offal,—were not washed, nor the hairs removed from the heads or feet. The health officer alluded to above writing in the *Lancet and Clinic* more than intimates that condemned hogs had been slaughtered and rendered into lard—'guts' and all; and even says, that hogs dead of *hog cholera*, (which is septi-cœmia, blood poison,—think of filth, so filthy as to poison the blood of a hog!)—had to be guarded at night by a strong police force, to prevent their being carted off by the distillery men, and *made into lard!*—'Think of it, dissolute man,' and then—eat of it, 'if you can!

"These reflections have been suggested by the recent reading of an account of the investigation referrered to, and the testimony of one James Mathews in particular, together with the article in the *Cincinnati Lancet*.'

"It seems to us that sanitarians and health authorities are diligently stopping the cracks and crevices—and leaving wide open the flood gates of disease. The sale of lard made as disclosed should be prohibited by heavy penalty, and the man or men who offer lard made from hogs, dead of disease, or diseased, should *be put to death*, as surely as the murderer who takes life by swifter, (but no surer,) means. The eating of hog's flesh, of any part of the swine, should be suppressed on sanitary grounds,—and, as to

*lard*, some vegetable substances could, and should be found. It has been found, and exists in abundance, right in our midst; and if the South had any enterprise, she could make it a source of immense revenue. We refer to cotton seed oil, a pure vegetable oil, as pure, and equally as good, as the oil of the olive, or the palm, and much cheaper ; in fact, the *time will come when cotton will be raised more for the oil than the lint.* Few know it, but the oil has already taken the place of olvie oil in the sardine bnsiness to a very large extent,—the bulk of export oil going to Italy and Constantinople.

"A crown of glory awaits the man or men who succeed in popularizing cotton seed oil or any other vegetable oil, as substitute for the villainous, filthy, dangerous, adulterated, unclean, unwholesome and God-cursed fat of the scavenger hog !"

